# In-situ monitoring for liquid metal jetting using a millimeter-wave impedance diagnostic

**DOI:** 10.1038/s41598-020-79266-2

**Published:** 2020-12-18

**Authors:** Tammy Chang, Saptarshi Mukherjee, Nicholas N. Watkins, David M. Stobbe, Owen Mays, Emer V. Baluyot, Andrew J. Pascall, Joseph W. Tringe

**Affiliations:** Lawrence Livermore National Laboratory, Livermore, CA 94550 USA

**Keywords:** Electrical and electronic engineering, Materials science, Mechanical engineering

## Abstract

This article presents a millimeter-wave diagnostic for the in-situ monitoring of liquid metal jetting additive manufacturing systems. The diagnostic leverages a T-junction waveguide device to monitor impedance changes due to jetted metal droplets in real time. An analytical formulation for the time-domain T-junction operation is presented and supported with a quasi-static full-wave electromagnetic simulation model. The approach is evaluated experimentally with metallic spheres of known diameters ranging from 0.79 to 3.18 mm. It is then demonstrated in a custom drop-on-demand liquid metal jetting system where effective droplet diameters ranging from 0.8 to 1.6 mm are detected. Experimental results demonstrate that this approach can provide information about droplet size, timing, and motion by monitoring a single parameter, the reflection coefficient amplitude at the input port. These results show the promise of the impedance diagnostic as a reliable in-situ characterization method for metal droplets in an advanced manufacturing system.

## Introduction

Recent progress in additive manufacturing has enabled the construction of geometrically complex structures from a wide range of materials^1–5^. For the additive manufacturing of metals, jetting-based approaches have been investigated as a cost-effective alternative to selective laser-melting techniques^6–8^. Liquid metal jetting also offers additional merits when compared with selective laser-melting such as reductions in oxide inclusions, voids and pores, and residual stress, as well as the avoidance of management and waste associated with metal powder beds. One primary approach for liquid metal jetting is drop-on-demand, which produces discrete droplets by inducing a volumetric change in the liquid metal via displacement of a piezoelectric material^6,9,10^, applied pressure^8,11,12^, or applied electromagnetic fields^13–15^. Because the quality of the jetted liquid metal droplets is influenced by many factors including driving voltage, pressure, atmosphere, liquid viscosity, material density, and surface tension, there is a need for in-situ diagnostics to ensure the print quality of the liquid metal droplets over the duration of the build process^16–18^. In particular, key parameters of interest for these diagnostics are droplet size, timing, and temperature.

Currently, high-speed videography has been the prevalent in-situ technique used to monitor droplet performance in jetting-based systems. Wang et al. demonstrated the use of a closed-loop control framework in which the driving voltage of an alternating magnetic field is tuned using optical data, however the authors acknowledged that the online processing speed in their approach needs to be improved^16^. Recently, Huang et al. have also demonstrated the use of unsupervised learning to predict droplet behavior and infer material properties, but monitoring has yet to be demonstrated in real-time^17^. Both works present compelling evidence for the use of image-based processing for droplet monitoring. However, over the course of a build process, high speed video data quickly scales to very large files, thereby introducing a major bottleneck for real-time processing. As such, this work investigates the utility of a radio frequency approach wherein droplet information may be inferred from microwave waveforms, which offer substantially lower dimensionality than that of optical diagnostics and would thereby significantly reduce the processing load.

While a microwave approach offers potential advantages, prior use of in-situ microwave diagnostics for jetting-based additive manufacturing is limited. Still, some microwave approaches have been utilized for the in-situ material characterization of small sub-centimeter scale objects. These approaches largely comprise two techniques: free-space transmission and resonance-based monitoring^19–22^. Free-space transmission schemes have been applied at microwave frequencies for grain and seed moisture characterization^21,23^. These techniques measure dielectric properties in bulk, rather than for single quantities. Similarly, resonant approaches have been utilized for the measurement of bulk solid and liquid properties^22,24,25^. Similar to the jetted metal droplet application, a cavity resonance approach was applied to the material characterization of free falling aqueous droplets, where shifts in resonance were used to determine the dielectric material properties of the falling liquid droplets^26^. However, this measurement technique requires multiple droplet measurements in order to determine the precise resonant frequency shift, again resulting in a measurement of bulk, not instantaneous, properties.

**Figure 1:**
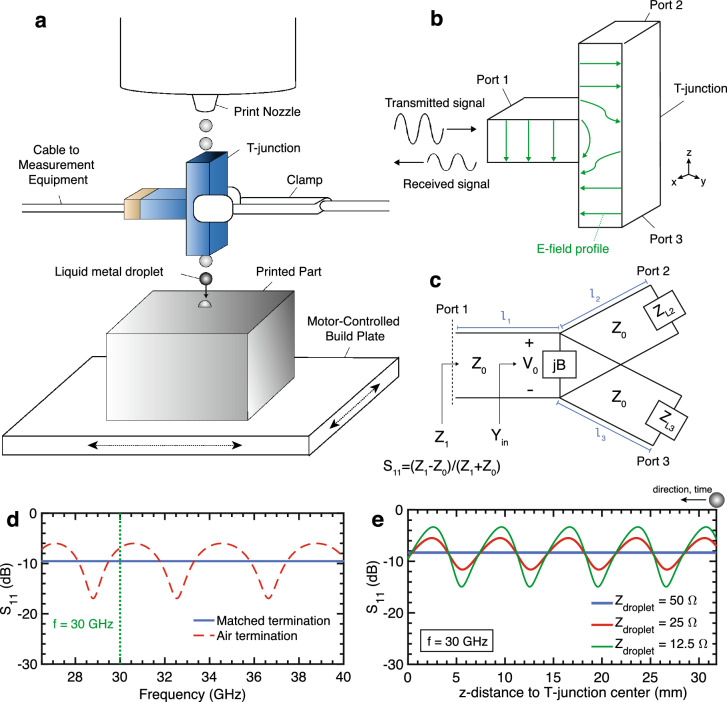
Open-ended T-junction as an impedance diagnostic for droplets in motion. (**a**) Implementation of an open-ended T-junction as an impedance diagnostic for liquid metal jetting. (**b**) The T-junction as a power divider, where port 1 is the input port and ports 2 and 3 are the output ports and are impedance matched. (**c**) Transmission line model of a lossless T-junction. The stored energy associated with the discontinuity of the junction is accounted for with susceptance *B*. (**d**) Analytical results for the return loss *S*_11_ when ports are terminated with a matched impedance and open air. (**e**) Analytical variation in *S*_11_ at f = 30 GHz [marked in (**d**)] for different droplet impedances. Note that symmetry is inherent in the T-junction and therefore only half of the T-junction length is shown.

**Figure 2:**
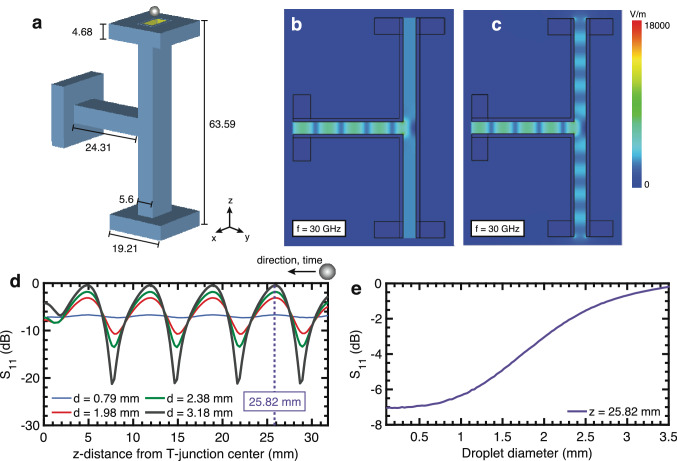
Open-ended T-junction simulation. (**a**) WR-28 waveguide T-junction model in simulation. Dimensions are in mm. (**b**) Time-averaged electric fields when port 2 and 3 are terminated with matched impedances (**c**) Time-averaged electric fields for open air terminations at port 2 and 3. (**d**) Return loss *S*_11_ at port 1 for droplets of four diameters (0.79, 1.98, 2.38, 3.18 mm) as the droplet moves from the edge of the T-junction arm to the center. Note that symmetry is inherent in the T-junction and therefore only half of the T-junction length is shown. (**e**) *S*_11_ when an aluminum sphere is positioned at z = 25.82 mm [marked in (**d**)]. The diameter is varied in 30 μm steps from 0.1 to 3.5 mm.

In this work, an in-situ impedance diagnostic is presented for the real-time monitoring of additive processes which employ liquid metal jetting. A waveguide T-junction with unterminated lateral ports is placed in line with the droplet stream (Fig. 1a). As consecutive droplets fall through the lateral path between port 2 and port 3, they cause a resulting impedance change to propagating signals in the waveguide. As a result, the droplets induce variations in the reflected signal as a function of time, which can be detected by monitoring the return loss *S*_11_ at a vector network analyzer (VNA) connected to port 1. Because this impedance approach is propagation-based, it has the ability to independently evaluate individual droplets, and thereby extract droplet dynamics and shape properties. It can also be scaled more practically to small droplet sizes due to the availability of required components at millimeter-wave frequencies. While preliminary results have been presented^27^, this work provides a comprehensive analysis of the diagnostic, from theory to experimental analysis.

In the following report, an analytical formulation is presented, using transmission line theory for the operation of the T-junction as a time-domain diagnostic. Next, a full-wave simulation model is built to validate the analytical formulation. Experimental validation is performed in controlled experiments with metallic spheres of known diameters. Finally, the diagnostic is demonstrated in a drop-on-demand liquid metal jetting system.

## Background and theory

The T-junction, a three-port microwave device, is conventionally used as a power divider^28–31^. When a signal is input at port 1 of an E-type rectangular waveguide T-junction, it produces outputs at ports 2 and 3 that are 180° out-of-phase (Fig. 1b). The amplitude of the output signals at port 2 and 3 can be determined using a transmission line model of a T-junction (Fig. 1c),

1 $$\begin{aligned} Y_{in}&= \frac{1}{Z_{2}} + \frac{1}{Z_{3}} + jB \end{aligned}$$

2 $$\begin{aligned} Z_{in}&= \frac{1}{Y_{in}} \end{aligned}$$

where $Z_{2}$ and $Z_{3}$ are the input impedances looking into port 2 and port 3, respectively, and B is the susceptance associated with fringe fields and higher order modes associated with the discontinuity at the junction. The input impedance $Z_{1}$ and reflection coefficient $\Gamma _{1}$ at port 1 are thus given by

3 $$\begin{aligned} Z_{1}&= Z_{0} \frac{Z_{in}+jZ_{0} \tan {\beta l_{1}}}{Z_{0}+j Z_{in} \tan {\beta l_{1}}} \end{aligned}$$

4 $$\begin{aligned} \Gamma _{1}&= \frac{Z_{1}-Z_{0}}{Z_{1}+Z_{0}} \end{aligned}$$

where $Z_{0} = 50~\Omega$, $\beta = 2\pi / \lambda$, and $\lambda$ is the wavelength of transmitted signal, $l_{1}$ is the length of the T-junction arm towards port 1, and the return loss $S_{11} = -20 \log _{10} |\Gamma _{1}|$.

The output ports of the T-junction are typically terminated with a matched characteristic impedance, often a rectangular waveguide of the same dimensions, such that $Z_{L2} = Z_{L3} = Z_{0}$. However, the impedance diagnostic presented in this report has open air terminations at ports 2 and 3 to allow the droplet stream to pass through the junction (Fig. 1a). Thus, $Z_{L2} = Z_{L3} = Z_{air}$, where $Z_{air}$ is the characteristic impedance of air at the boundary of the waveguide edge. Because there is no longer matching between the waveguide and load impedance, in accordance with transmission line theory, $Z_{2}$ and $Z_{3}$ are now wavelength-dependent and expressed as

5 $$\begin{aligned} Z_{2}&= Z_{0} \frac{Z_{air}+jZ_{0} \tan (\beta l_{2})}{Z_{0}+jZ_{air} \tan (\beta l_{2})} \end{aligned}$$

6 $$\begin{aligned} Z_{3}&= Z_{0} \frac{Z_{air}+jZ_{0} \tan (\beta l_{3})}{Z_{0}+jZ_{air} \tan (\beta l_{3})} \end{aligned}$$

where $l_{2}$ and $l_{3}$ are the lengths of the T-junction arms. The return loss of the T-junction under static conditions can be determined using Eqs. (1) – (4) and is shown in Fig. 1d. Analytical results for return loss across frequency show that when ports 2 and 3 are no longer terminated with matched impedances, the mismatch between $Z_{0}$ and $Z_{air}$ produces a response that is frequency-dependent (Fig. 1d).

When a droplet enters port 2, the assumptions that $Z_{L2} = Z_{air}$ and $l = l_{2}$ no longer hold. The metal droplet produces a reflective boundary which can be approximated by associating an impedance $Z_{d} = R_{d} + jX_{d}$, where $R_{d}$ represents dissipated energy and $X_{d}$ represents stored energy (capacitive or inductive) near the droplet location. Additional discussion on this approximation is included in the Methods section. In addition to the change in the value of the impedance termination, the droplet’s location is dynamic as it enters and moves through arm 2 of the T-junction, whereby *l* decreases as a function of time. In this scenario, the droplet location is represented by *l*(*t*) and:

7 $$\begin{aligned} {Z_{2}}(t)&= Z_{0} \frac{Z_{d}+jZ_{0} \tan (\beta l(t))}{Z_{0}+jZ_{d} \tan (\beta l(t))} \end{aligned}$$

8 $$\begin{aligned} Z_{3}&= Z_{0} \frac{Z_{air}+jZ_{0} \tan (\beta l_{3})}{Z_{0}+jZ_{air} \tan (\beta l_{3})} \end{aligned}$$

until the droplet enters the center area of the T-junction and into the bottom arm (leading to port 3), at which point $Z_{2}$ becomes static and ${Z_{3}}(t)$ becomes time-dependent.

The T-junction can thus capture time-domain characteristics of liquid metal droplets through the measurement of ${S_{11}}(t)$, which captures time-varying responses in ${Z_{2}}(t)$ and ${Z_{3}}(t)$. This relationship is shown in Fig. 1d–e, and is clearly demonstrated when the variation in *S*_11_ amplitude at a single frequency (e.g., $f = 30\,{\text{GHz}}$) is plotted over time. As the droplet location *l*(*t*) is varied, *S*_11_ varies periodically with droplet location. Furthermore, the resulting characteristics of the waveform vary with the real (Fig. 1e) and imaginary (Supplementary Fig. S1) values of the impedance $Z_{d}$. As is clear from the geometry of the system, the symmetry of the T-junction implies a symmetric response at locations on either side of the T-junction center.

## Results

### Simulation

To further understand the implications of the analytical open-ended T-junction model as it relates to droplet size and dynamics, electromagnetic full-wave simulations were conducted. A representative T-junction was constructed to match an experimental device (Fig. 2a). Details of the simulation setup are provided in the Methods section. The time-averaged results are shown for matched (Fig. 2b) and open terminations (Fig. 2c) at 30 GHz. While constant electromagnetic propagation is observed out of the arm of port 2 and port 3 in the matched case, field reflections due to the open termination produce standing waves across vertical arms of the T-junction. The nodes and antinodes of these standing waves then dictate the resulting *S*_11_ waveform when a droplet enters the T-junction.

When a spherical metallic droplet enters the T-junction from port 2, it creates a moving impedance discontinuity. For very small droplets, the perturbation is minimal due to the small impedance discontinuity (Fig. 2d), and approaches the constant *S*_11_ value in the case when no droplet is present. However, as the droplet size increases, its effects are more prominent, most notably at nodes and antinodes of the standing wave. When the droplet diameter is close to the internal height of the waveguide ($h = 3.556$ mm), it is able to reflect a larger percentage of the outgoing signal at antinode locations, resulting in a near maximum *S*_11_ magnitude of 0 dB at peak locations in the waveform. These trends are also observed in Fig. 2e, where droplet diameter is varied while maintaining a fixed location within the waveguide at $z = 25.82$ mm. The relationship between droplet diameter and *S*_11_ magnitude is monotonic and shows characteristics of a sigmoid curve, where saturation is observed at smaller and larger diameters relative to the internal waveguide height. Trends observed in simulation show meaningful information can be extracted from measured millimeter-wave signals, and are evaluated experimentally in the following section.

**Figure 3:**
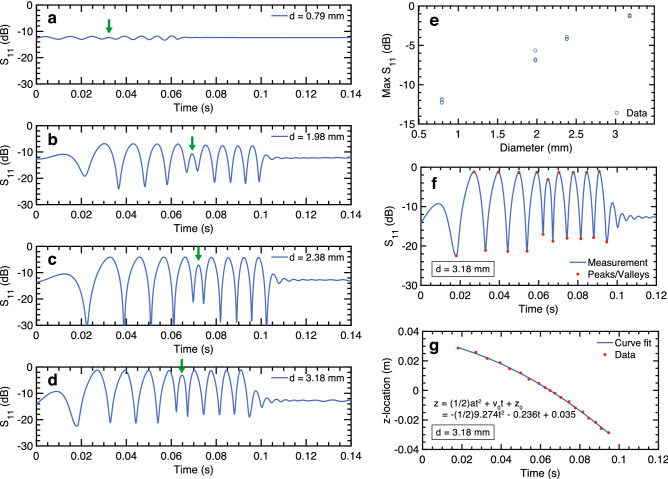
Solid metal sphere experiments. Time-domain *S*_11_ measurement for aluminum spheres with diameters: (**a**) 0.79 mm, (**b**) 1.98 mm, (**c**) 2.38 mm, (**d**) 3.18 mm. The center lobe that marks the center of the T-junction is denoted by a green arrow. (**e**) Peak *S*_11_ value (averaged over 8 peaks) for each sphere drop, as a function of sphere diameter. (**f**) Measured waveform for sphere with 3.18 mm diameter. Extrema are marked in red and can be associated with known positions in the T-junction. (**g**) The time stamps of extrema in (**f**) are plotted with position. A second-order polynomial curve fit is applied.

**Figure 4:**
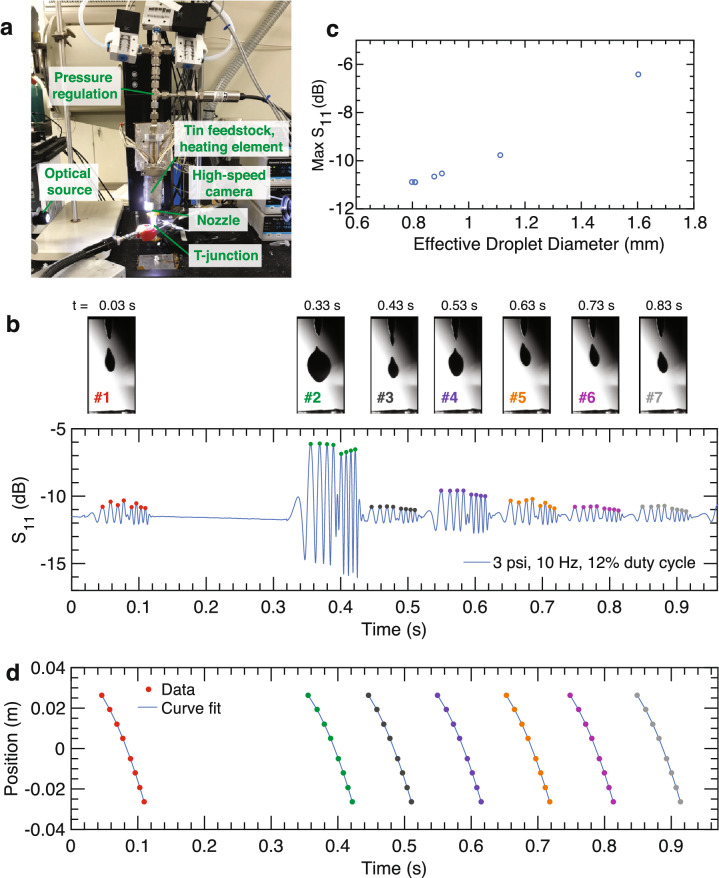
Liquid metal experiments. (**a**) Experimental setup in liquid metal jetting drop-on-demand system. (**b**) Time-domain *S*_11_ measurement on tin droplets for a 200 um nozzle with 10 Hz pressure pulses. Peak values are denoted with colored markers. Images of jetted droplets are shown above the corresponding *S*_11_ signal perturbation. (**c**) Peak *S*_11_ value (averaged over 8 peaks) for each droplet plotted as a function of effective droplet diameter. (**d**) Droplet position (height, z-axis) over time for each droplet. Colored markers correspond to known peak locations and curves (blue) correspond to a second-order polynomial curve fit. The constants yielded by the curve fit are provided in Table 1.

### Experiment

#### Solid metal spheres

The impedance diagnostic was first evaluated in a controlled experiment with four aluminum spheres of known diameters (0.79, 1.98, 2.38, 3.18 mm). The experimental setup is shown in Supplementary Fig. S3. The measured time-domain signals for each sphere are shown in Fig. 3a–d. The measured *S*_11_ signal variation exhibits the same features as those predicted by simulation for a spherical conductor in an open-ended T-junction (Fig. 2d). Four side lobes are observed for each time-domain *S*_11_ waveform on each side of a lower center lobe, and correspond to field perturbation as the spheres travel through standing wave locations in the T-junction. Acceleration due to gravity leads to progressively narrower lobes, producing an asymmetric result between eaach side of the center lobe. The result in Fig. 3a required a manual trigger which resulted in a time offset in waveform capture, hence the first side lobe was not captured.

Similar to what is observed in Fig. 2d, the center lobe is lower and its corresponding nulls are higher than the surrounding side lobes and their corresponding nulls. These differences are attributed to side wall coupling near the center of the T-junction and a difference in droplet reflection characteristics at the center of the T-junction, when compared to reflection in the T-junction arms.

As with the simulation results, there is an observable increase in the *S*_11_ magnitude at the peaks of the lobes as the sphere diameter is increased in Fig. 3a–d. The average of peak *S*_11_ values was calculated for each captured dataset and shows a sparse monotonic profile with sphere diameter (Fig. 3e). This result indicates that waveform amplitude can be correlated with sphere size. The larger variation in average amplitude in the 1.98 mm results in Fig. 3e is attributed to variation in the x and y location of the spheres as they fall through the T-junction, which in some cases leads to a higher single peak value in time.

In addition to associated peak amplitude with sphere size, the time stamps for the extrema in the waveform can be used to track the position of the sphere within the waveguide as a function of time (Fig. 3f,g). By associating maxima and minima values with known locations in the waveguide (for details, see Methods section), the effective droplet dynamics can be extrapolated. The data is fit to a constant acceleration model where $z(t) = \frac{1}{2}a t^2 + v_{0} t + z_{0}$ and $v(t) = a t + v_{0}$. For the dataset plotted in Fig. 3, the acceleration is $-9.27$ m s^−1^, close to the value for free fall ($-\,9.81$ m s^−1^).

#### Liquid metal jetting

Following evaluation in the controlled environment, the impedance diagnostic was evaluated in a custom-built liquid metal jetting, drop-on-demand system^8^ (Fig. 4a). This system uses a temperature-controlled melt chamber to melt 100% tin feedstock. Controlled pressure actuation is employed to deposit liquid tin droplets. Details of the print system and settings are provided in the Methods section. The T-junction was aligned with the droplet stream and operated with a continuous-wave, 29 GHz signal. High-resolution video was taken during the experiment for comparison and video frames are shown of the droplets before they enter the T-junction. These images are shown with the corresponding millimeter-wave signal perturbation produced by the droplet as it passes through the T-junction for a 10 Hz pressure signal (Fig. 4b).

The droplets produce similar waveform profiles to those of the solid sphere experiments. Several peaks are present on both sides of a center lobe for each droplet waveform. It is also evident that the variation in droplet size corresponds to a change in the peak magnitude of the measured signal. Additionally, the captured millimeter-wave signal indicates that between $t = 130$ ms and $t = 330$ ms, no droplets were jetted, despite the pressure excitation. Instead, a larger cumulative droplet is jetted at $t = 330$ ms. Image analysis was conducted on the droplets (Supplementary Fig. S6, Table S2) to determine effective droplet diameter from the two-dimensional droplet area. These results are plotted with the associated return loss (Fig. 4c) and show a monotonic increase in *S*_11_ with effective diameter. Additional results also suggest that shape features can be captured by this diagnostic (Supplementary Fig. S7).

**Table 1:** Results of second-order polynomial curve fit for experimental results in Fig. 4d based on the constant acceleration model $z(t) = \frac{1}{2}a t^2 + v_{0} t + z_{0}$.

Droplet #	$a$ (m s^−2^)	$v_{0}$ (m s^−1^)
1	− 9.654 ± 0.201	− 0.522 ± 0.011
2	− 9.804 ± 0.174	− 0.469 ± 0.010
3	− 9.637 ± 0.185	− 0.508 ± 0.010
4	− 9.805 ± 0.202	− 0.482 ± 0.011
5	− 9.422 ± 0.217	− 0.504 ± 0.011
6	− 9.707 ± 0.195	− 0.498 ± 0.011
7	− 9.754 ± 0.159	− 0.495 ± 0.009

Results are given with 95% confident interval bounds. $z_{0} = 0.026$ in all cases.

The extrema of the waveforms in Fig. 4b were associated with the corresponding time stamps for each peak in the captured waveform (Fig. 4d). A second-order polynomial curve fit yields the effective dynamics of the jetted droplets as they travel through the T-junction (Table 1). The results show a smaller confidence interval, and therefore higher precision of the extracted velocity values, compared with acceleration. Initial velocities as droplets enter the T-junction are on the order of $-0.5$ m s^−1^ and t = 0 represents the first peak in the T-junction ($z = 26.34$ mm).

## Discussion

The results herein show promise for the use of a millimeter-wave diagnostic, based on an open-ended T-junction structure, to capture the characteristics of jetted metal droplets in real-time. This approach can be applied more generally to non-metallic materials as well, although the sensitivity of the measurement remains to be investigated. Although this paper focuses on establishing dimensional aspects of jetted or falling droplets, it is also possible to extract material properties such as conductivity, which would provide important insight about corresponding droplet temperature. However, material properties need to be decoupled from effects due to the size or shape of the droplets. An inverse modeling approach could help to evaluate these distinctions. We also note that to achieve sensitivity for smaller droplet sizes on the order of several hundred microns, this same setup can be scaled for operation at higher frequencies. In this case, a smaller waveguide size would be used to achieve higher frequency propagation (Supplementary Fig. S8).

There are also additional opportunities to further develop this diagnostic. Namely, shortening the vertical T-junction arms would reduce the distance between the nozzle and build plate, and also allow for an increased droplet print rate, at the cost of reduced data points to determine individual droplet dynamics. Additionally, the magnitude of *S*_11_ has been the focus of this work, but a detailed investigation of *S*_11_ phase variation would offer a more comprehensive picture of how the transmitted signal is perturbed by the presence of the droplet.

Finally, while the droplet size assessment in this paper is shown for droplets of similar size and nearly spherical shapes, this may not always be the case if an oxide layer forms around the droplet during jetting. To account for shape variation analytically, more rigorous development is required to account for wave scattering mechanisms associated with droplets of different shapes. Sparse correlation techniques between video images and microwave signals could also bridge this modeling gap and be used to establish an efficient, real-time control loop for liquid metal jetting systems.

## Methods

### Transmission line model

For simplicity, the droplet approximation in this work assumes the droplet acts as a lossy, reflective boundary at its location in the waveguide. More rigorous full-wave scattering formulations for a conducting sphere in a rectangular waveguide are provided in prior literature^32–35^.

### Electromagnetic simulation

A commercial full-wave electromagnetic solver was used for simulation. The simulated metal spheres are aluminum ($\sigma = 3.56 \times 10^{7}$ S m^−1^) and the T-junction is copper ($\sigma = 5.8 \times 10^{7}$ S m^−1^). Waveguide dimensions are standard WR-28, $w = 7.112$ mm and $h = 3.556$ mm. Other dimensions are shown in Fig. 2a and based on the commercial WR-28 T-junction (Microwave Associates, MA-560) used in experiment. The diameter of the metal sphere at $z = 25.82$ mm was varied in 30 μm steps to produce the curve in Fig. 2e. It is evident that at smaller droplet diameters around 0.5 mm, the change in *S*_11_ becomes small and, as a result, the *S*_11_ variation due the falling droplet becomes difficult to detect (Supplementary Fig. S2). Droplet resolution based on the sigmoid curve is also shown in Fig. S2.

### Solid metal sphere experiment

The solid metal sphere setup utilizes a microfunnel to align the sphere trajectory with the T-junction (Supplementary Fig. S3) . Aluminum ball bearings were used. An infrared photogate sensor (880 nm wavelength) was used to trigger the Vector Network Analyzer to conduct a continuous-wave measurement (f = 30 GHz) as the sphere enters the T-junction. In order to achieve a sufficiently fast sampling rate, the VNA is configured for a single frequency, time-domain measurement of return loss. To validate the extrema locations as a sphere passed through the waveguide, a controlled experiment was constructed with a programmable linear stage to vary the location of the sphere in the T-junction (Supplementary Fig. S4, Table S1). The presence of a second sphere in the T-junction before the first sphere exits was also characterized (Fig. S5), and it is demonstrated that the presence of multiple droplets in the T-junction can have a distinct and identifiable waveform from the presence of a single droplet.

### Liquid metal jetting experiment

The jetting system heats 100% tin feedstock (melting temperature 232°) in a temperature-controlled melt chamber at 340°, and a custom Matlab program is used to control the droplet frequency and velocity using electronically-actuated pressure pulses. The waveform in Fig. 4b was measured for a 200 μm inner diameter nozzle (300 μm outer diameter) with 20.68 kPa pulses triggered electronically at 10 Hz and 12% duty cycle. Signals captured with different print settings are shown in Supplementary Fig. S7. The liquid metal jetting was performed in an open air environment that leads to the formation of a surface oxide, which results in irregular droplet shapes. Using an inert gas environment (e.g., argon) can produce consistently uniform, spherical droplets. A polyimide lining (140 μm thick) was inserted into the T-junction to protect the waveguide walls from tin droplets. The frequency selection was lowered to 29 GHz for comparability due to the dielectric loading effect of the polyimide lining.

## Supplementary information

Supplementary Information.
