# Supplementary material for: In-situ monitoring for liquid metal jetting using a millimeter-wave impedance diagnostic
Source: Sci Rep. 2020 Dec 18;10:22325. doi: 10.1038/s41598-020-79266-2 (PMC7749153; doi:10.1038/s41598-020-79266-2)
Supplement: Supplementary file 1 — Supplementary Information. [file 41598_2020_79266_MOESM1_ESM.pdf]

Supplementary Information

In-Situ Monitoring for Liquid Metal Jetting Using a Millimeter-Wave Impedance Diagnostic

Authors: Tammy Chang, Saptarshi Mukherjee, Nicholas N. Watkins, David M. Stobbe, Owen Mays, Emer V. Baluyot, Andrew J. Pascall, Joseph W. Tringe

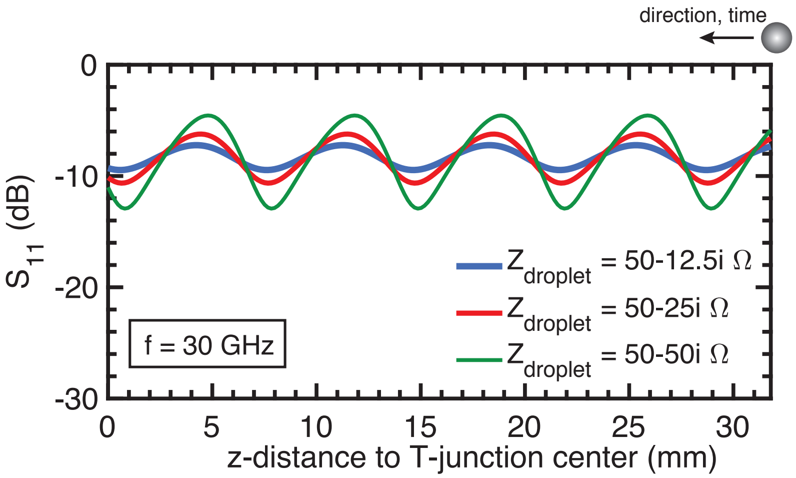

Figure S1. Analytical variation in S11 at f = 30 GHz for different complex droplet impedances.

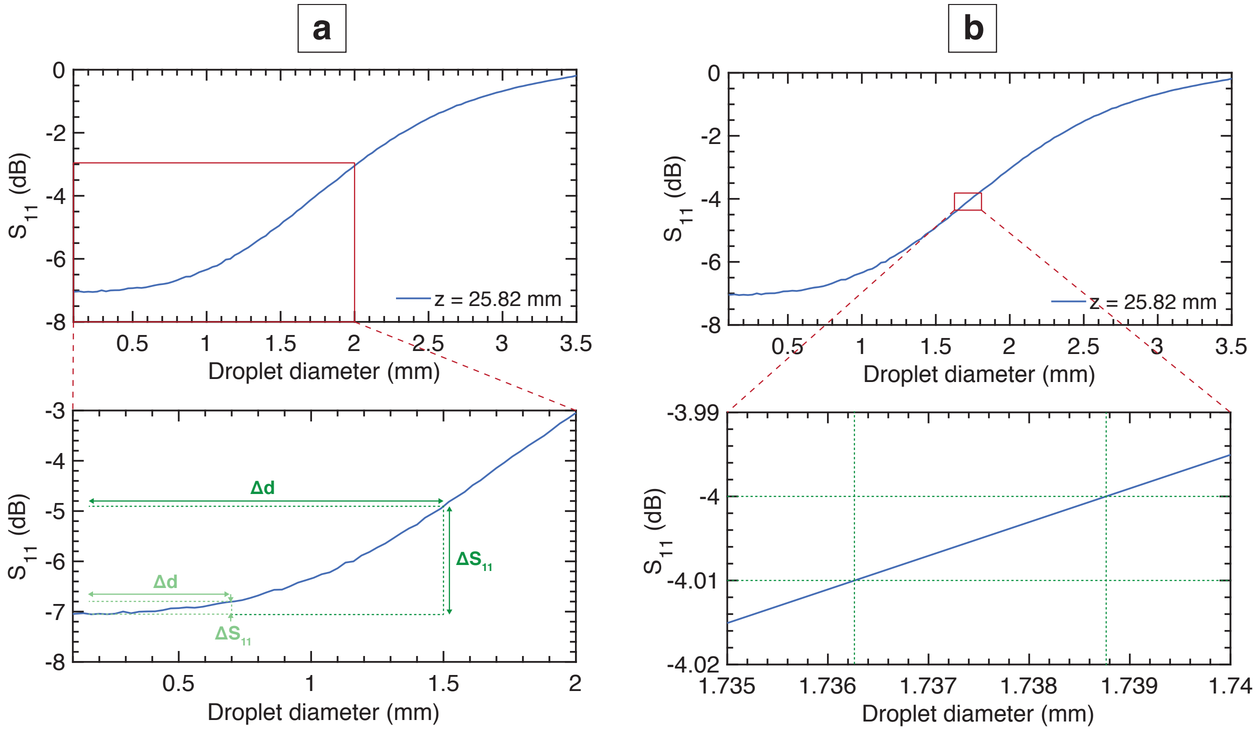

Figure S2. Simulated sensitivity and resolution for different droplet sizes at a fixed location (z = 25.82 mm) and frequency (f = 30 GHz). (a) Droplets with 1.5 mm diameter will yield a larger change in S11 than droplets diameters 0.75 mm. These results point to a lower detection limit around 0.5 mm. (b) The detectable droplet size variation depends on the VNA resolution. For a 0.01 dB resolution, the droplet diameter step variation is 2.6 um. Exact values for droplet sensitivity and size resolvability depend on the resolution and noise level of the vector network analyzer.

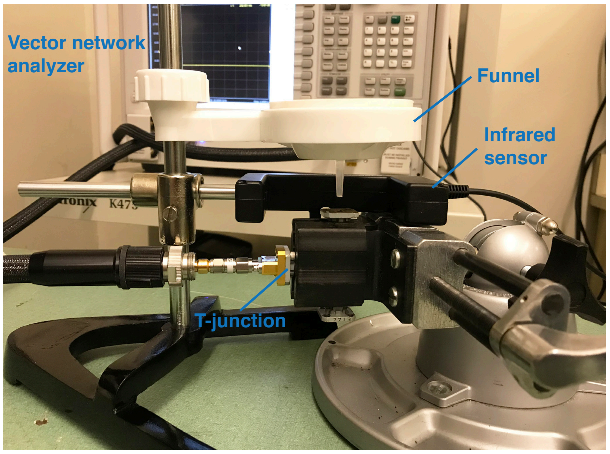

Figure S3. Solid metal sphere experimental setup. A funnel is used to direct metal spheres into the open-ended T-junction. A vector network analyzer is triggered by the infrared sensor.

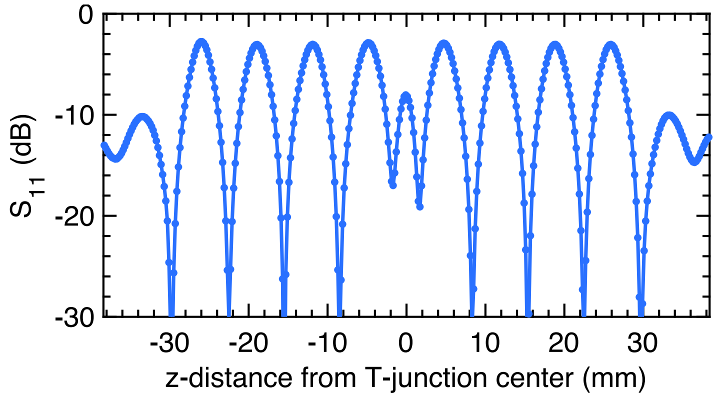

Figure S4. Measured waveform for varying location of metal sphere in T-junction, in 0.2 mm steps, for a 2.38 mm diameter sphere at f = 30 GHz.

Table S1. Peak locations in simulation (step size = 0.26 mm) and experiment (step size = 0.2 mm). The mean error is 0.095 mm.

Peaks (Sim.) [mm]	Peaks (Exp.) [mm]	Δ = |Peaks (Exp.) – Peaks (Sim.)| [mm]
-25.90	-25.82	0.079
-18.90	-18.80	0.099
-11.90	-11.78	0.119
-4.90	-4.76	0.139
-0.10	0.00	0.099
4.70	4.76	0.059
11.70	11.78	0.079
18.90	18.80	0.101
25.90	25.82	0.081

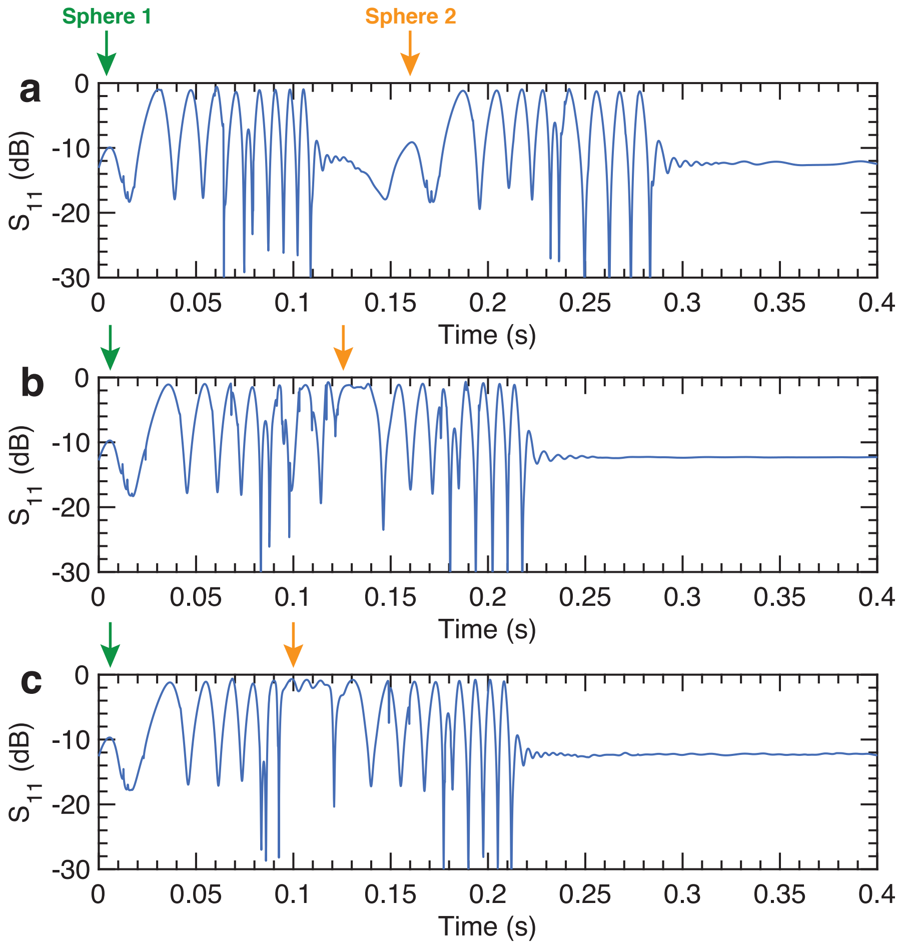

Figure S5. Measured time-domain waveform when f = 30 GHz for two spheres (3.18 mm diameter) falling through T-junction. Green and orange arrows are used to denote the time when sphere 1 and 2 enter the T-junction, respectively. (a) The spheres are dropped consecutively, as evidenced by the distinct waveform profile for each droplet. (b)-(c) Two spheres enter the T-junction consecutively. The overlapping time period can be determined based on when the flat top waveform appears.

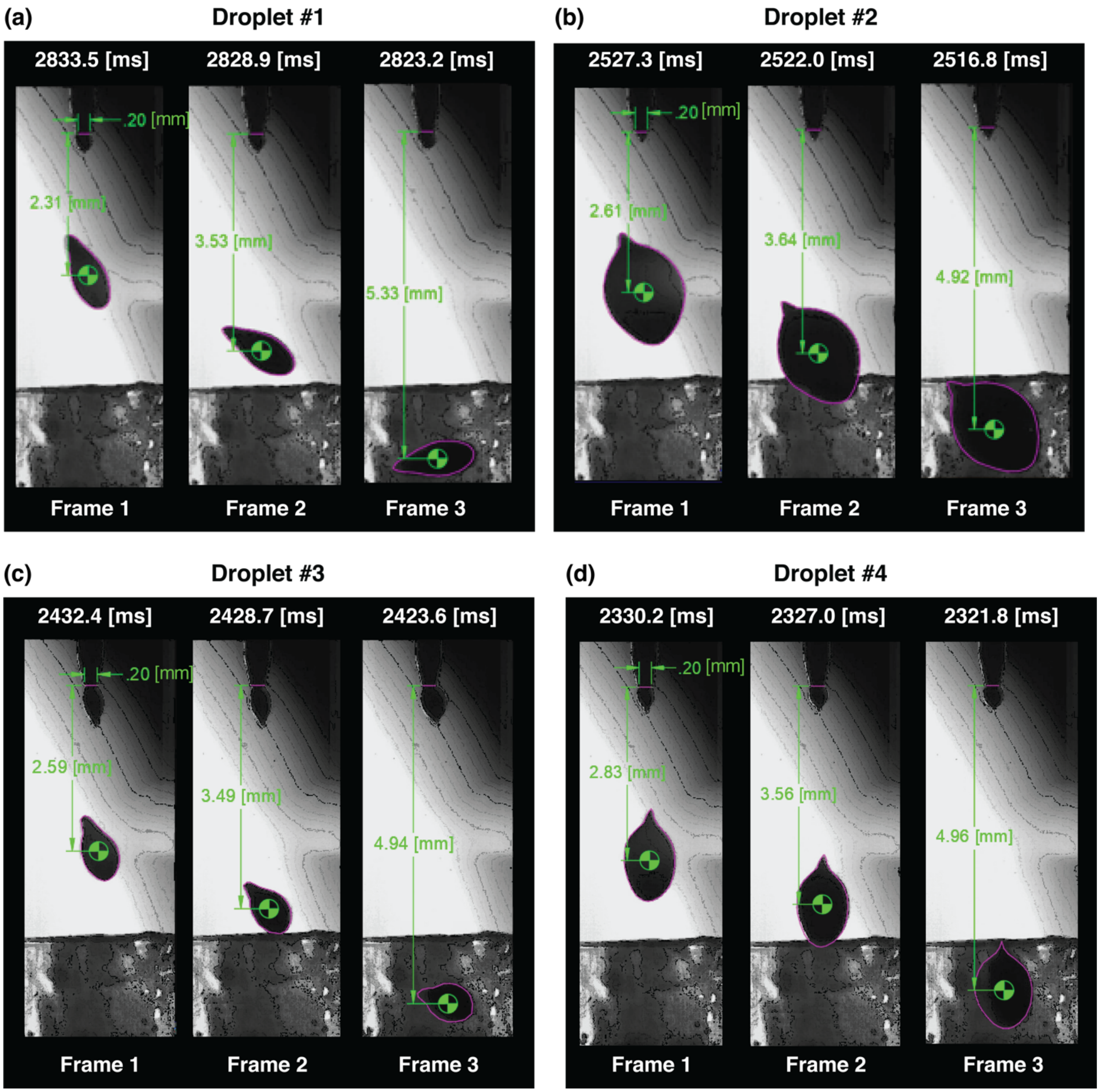

Figure S6. Applied image analysis to determine droplet area, velocity, and average acceleration for droplets #1-#4 in Fig. 4 results.

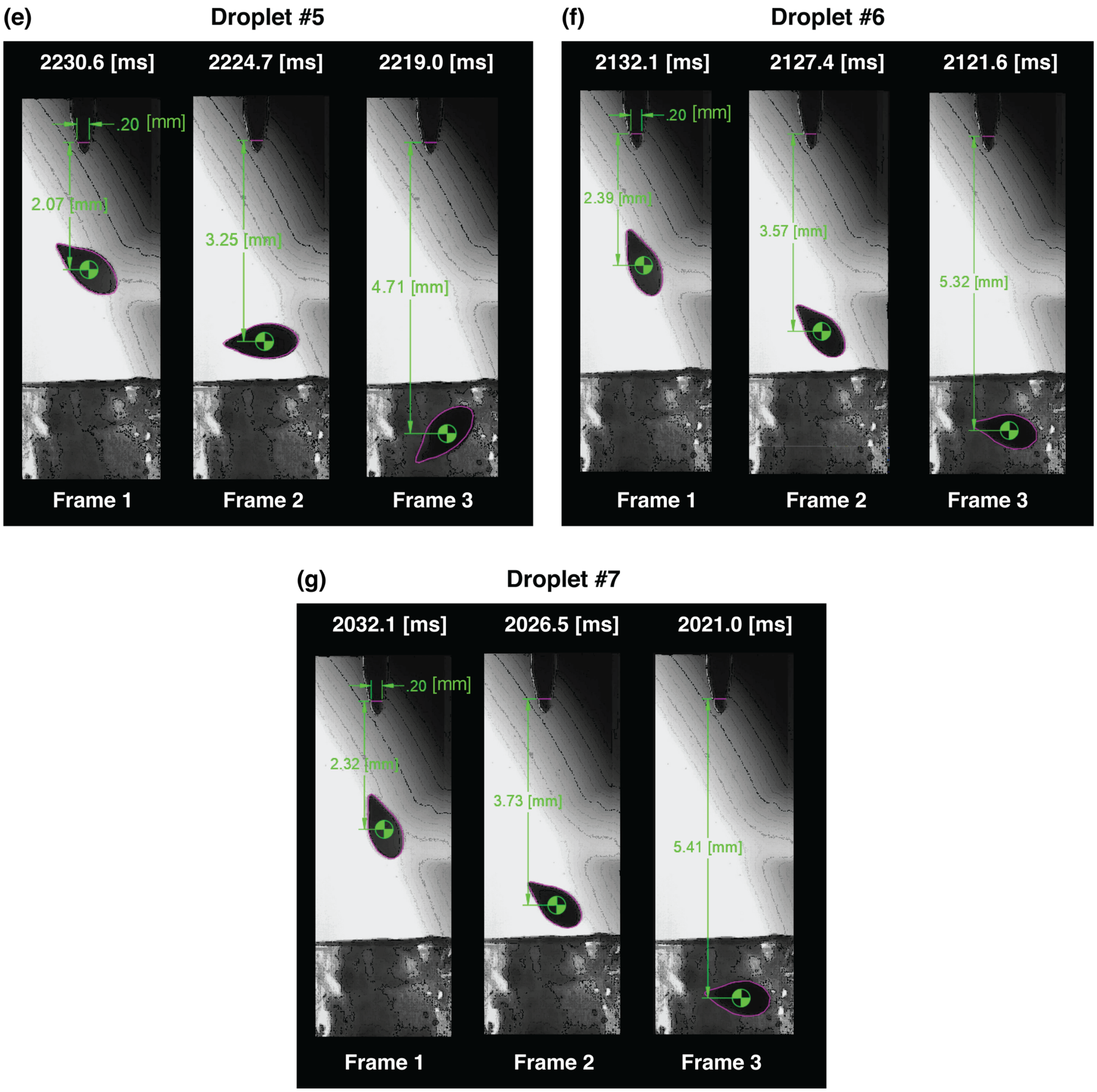

Figure S6 (cont'd). Applied image analysis to determine droplet area, velocity, and average acceleration for droplets #5-#7 in Fig. 4 results.

Table S2. Results of image analysis conducted on high-speed video images for data in Fig. 4. Frame numbers correspond to those shown in Fig. S4.

**Droplet**	**Area** Frame 3 [mm^2]	**Average Velocity** Frames 2-3 [m/s]	**Average Acceleration** Frames 1-3 [m/s^2]	**Predicted Velocity** t = 0, z = 26.34 mm **[**m/s]
1	0.605	-0.32	-9.82	-0.52
2	2.018	-0.25	-9.87	-0.47
3	0.501	-0.28	-9.33	-0.51
4	0.972	-0.27	-9.79	-0.48
5	0.643	-0.26	-9.68	-0.50
6	0.514	-0.30	-9.65	-0.50
7	0.516	-0.31	-9.67	-0.49

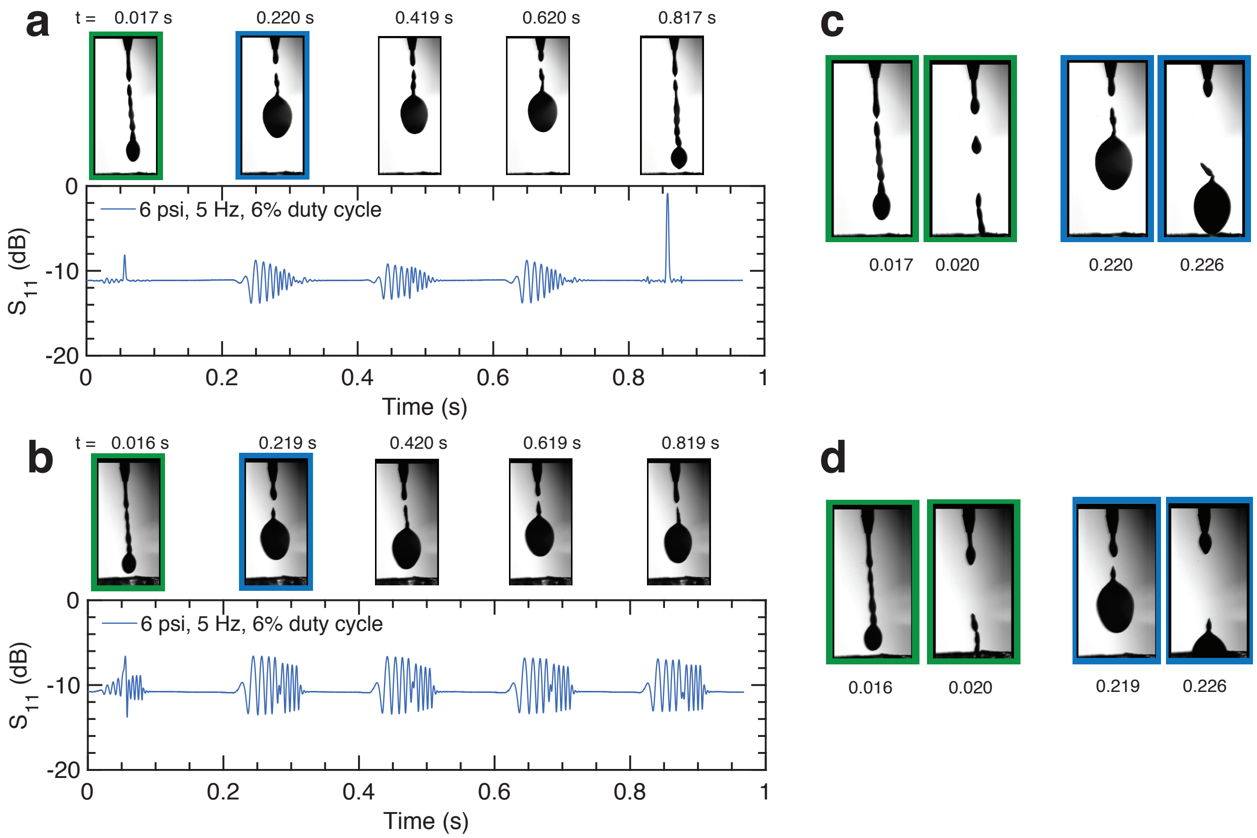

Figure S7. Results for 6 psi, 5 Hz, 6% duty cycle pressure pulses and 200 um inner diameter (300 µm outer diameter) nozzle. Although single video frames imply that the first and second droplets in (a) and (b) are the same, however, the microwave signal differs. Further investigation shows that there are distinct differences between these droplets as they enter the T-junction, as shown in (c) and (d).

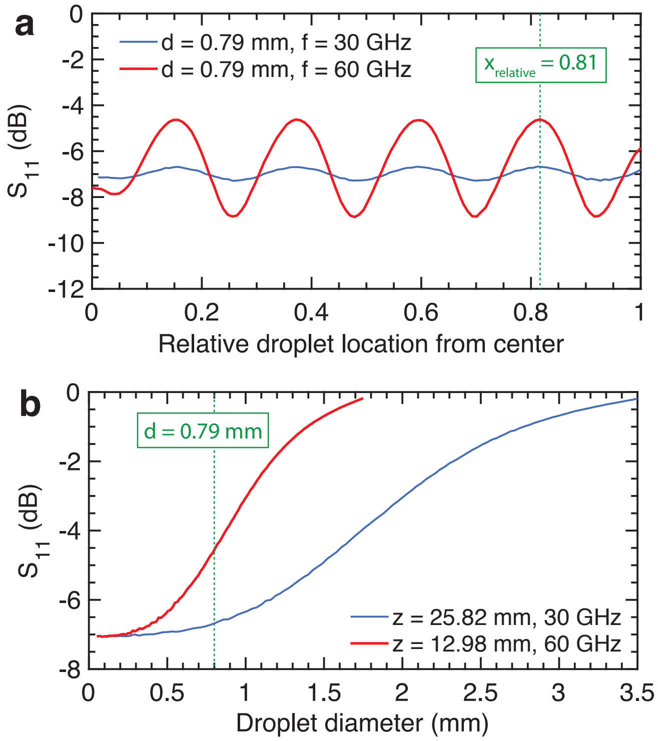

Figure S8. Comparison of simulated S_{11} variation at two frequencies (f = 30 GHz, 60 GHz) for: (a) a fixed droplet diameter (d = 0.79 mm) and varied location in the T-junction; (b) a fixed relative droplet location (0.81) and varied droplet diameter. The relative droplet location is the ratio between exact location in the T-junction and the length of the T-junction arm (31.8 mm for 30 GHz, 15.9 mm for 60 GHz). When $x_{relative}$ = 1, the droplet is located at the edge of the T-junction.
